# Examination of Akt and GSK3β in BDNF‐mediated reductions in BACE1 activity in neuronal cells

**DOI:** 10.14814/phy2.70001

**Published:** 2024-08-19

**Authors:** B. J. Baranowski, A. Mohammad, P. J. LeBlanc, V. A. Fajardo, R.E.K. MacPherson

**Affiliations:** ^1^ Department of Health Sciences Brock University St. Catharines Ontario Canada; ^2^ Department of Kinesiology Brock University St. Catharines Ontario Canada; ^3^ Centre for Neuroscience Brock University St. Catharines Ontario Canada

**Keywords:** BACE1, BDNF, GSK3B, SH‐SY5Y

## Abstract

Brain‐derived neurotrophic factor (BDNF) content and signaling has been identified as one potential regulator of amyloid precursor protein (APP) processing. Recently published work has demonstrated that BDNF reduces BACE1 activity while also elevating the inhibition of GSK3β in the prefrontal cortex of male C57BL/6J mice. These results provide evidence that BDNF alters APP processing by reducing BACE1 activity, which may act through GSK3β inhibition. The purpose of this study was to further explore the role of GSK3β in BDNF‐induced regulation on BACE1 activity. We utilized a cell culture and an in vitro activity assay model to pharmacologically target BDNF and GSK3β signaling to confirm its involvement in the BDNF response. Treatment of differentiated SH‐SY5Y neuronal cells with 75 ng/mL BDNF resulted in elevated pTrkB content, pAkt content, pGSK3β content, and reduced BACE1 activity. An in vitro BACE1 activity assay utilizing mouse prefrontal cortex (*n* = 6/group) supplemented with BDNF, BDNF + ANA12 (Trkb antagonist), or BDNF + wortmannin (Akt inhibitor) demonstrated that BDNF reduced BACE1 activity; however, in the presence of TrkB or Akt inhibition, this effect was abolished. An in vitro ADAM10 activity assay utilizing mouse prefrontal cortex (*n* = 6/group) supplemented with BDNF, BDNF + ANA12 (Trkb antagonist), or BDNF + wortmannin (Akt inhibitor) demonstrated that BDNF did not alter ADAM10 activity. However, inhibiting BDNF signaling reduced ADAM10 activity. Collectively these studies suggest that GSK3β inhibition may be necessary for BDNF‐induced reductions in BACE1 activity. These findings will allow for the optimization of future therapeutic strategies by selectively targeting TrkB activation and GSK3β inhibition.

## Graphical abstract

BDNF reduces BACE1 activity via Akt.

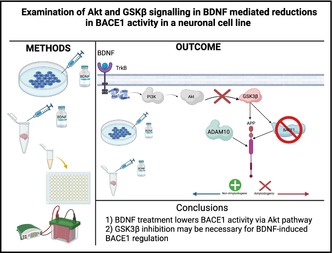

## 1 INTRODUCTION

Brain derived neurotrophic factor (BDNF), known for its role in synaptic plasticity, neurogenesis and neuron survival, has recently been found to play a role in the processing of the transmembrane protein amyloid precursor protein (APP) (Baranowski et al., 1985; Nigam et al., 2017). The fate of APP processing is determined by one of two competing pathways (Wang et al., 2018). The first pathway is initiated when the rate‐limiting enzyme, beta‐site amyloid precursor protein cleaving enzyme 1 (BACE1), cleaves APP resulting in the release of a soluble ectodomain, soluble APPβ (sAPPβ) fragment, and leaving behind a membrane‐bound C‐terminal fragment (CTF) 89/99 (Hardy & Higgins, 1992; Nunan & Small, 2000). ϒ‐Secretase then cleaves the CTF89/99 fragment and releases beta‐amyloid peptides (Aβ) from the membrane (Chow et al., 2010; Selkoe, 2001a, 2001b). Conversely, APP can be cleaved by ⍺‐secretase (ADAM10); however, ADAM10 cleaves APP within the Aβ domain, and the products of this pathway are considered non‐pathological (Endres & Fahrenholz, 2012). Previous work has demonstrated a potential link between BDNF and BACE1 and ADAM10 activity (Baranowski et al., 2021; Nigam et al., 2017), however the mechanisms underlying this connected remain unknown.

The accumulation of Aβ peptides is detrimental to neuronal networks and leads to neuronal death and dysfunction (Gouras et al., 2005). The mechanisms leading to the accumulation of these peptides are multifaceted. As BACE1 initiates APP processing leading to the production of Aβ peptides (Hampel & Shen, 2009; Hardy & Higgins, 1992; Vassar et al., 1999), it is essential to understand the mechanisms that regulate the activity of BACE1 in the brain. Evidence suggests that Aβ accumulation could be due to BDNF deficiency (Peng et al., 2009). To support the role of BDNF in APP processing, both human (post‐mortem brains of patients with Alzheimer's disease (Allen et al., 1999; Ferrer et al., 2000; Fumagalli et al., 2006; Hock et al., 2000; Holsinger et al., 2000; Phillips et al., 1991)) and rodent models (APP^NLh^/PS‐1^P264L^ and TgCRND8 (Peng et al., 2009)) in which Aβ accumulation occurs, display lower expression and protein content of BDNF and its receptor, tyrosine receptor kinase B (TrkB; also known as tropomyosin receptor kinase B). A strong correlation between Aβ oligomers build‐up in the frontoparietal cortex and lower BDNF mRNA levels have also been demonstrated further indicating a link between Aβ aggregation and lower BDNF levels (Peng et al., 2009). Our work has demonstrated that direct treatment of brain tissue explants with BDNF reduced BACE1 activity in the prefrontal cortex of male C57BL/6J mice (Baranowski et al., 2021). This demonstrates that BDNF may be important in altering APP processing by reducing BACE1 activity. Other work has demonstrated a connection between BDNF and ADAM10 in a neuronal cell line (Nigam et al., 2017). SH‐SY5Y neuronal cells treated with BDNF (50 ng/mL) reduced Aβ peptide production and increased sAPPα content, and this effect was abolished when these cells were treated with batimastat (an ADAM10 inhibitor) in combination with BDNF (Nigam et al., 2017). Together, this work indicates that BDNF may play a role in APP processing by regulating BACE1 and ADAM10, however, whether BDNF treatment could elicit these alterations in an in vivo model remained unexplored. Recently, our research aimed to determine whether chronic peripheral BDNF injections in an in vivo model could regulate the activity of APP processing enzymes and effect cognition and behavior (Baranowski et al., 1985). Chronic peripheral BDNF injections resulted in improved cognitive function (measured through active recognition memory) and a shift in APP processing towards the non‐amyloidogenic pathway. Specifically, peripheral BDNF injections reduced BACE1 activity and elevated ADAM10 activity, indicating a shift in APP processing. This work highlights the potential of BDNF treatment to alter APP processing through both BACE1 and ADAM10. Despite these novel findings, a direct link and mechanism by which BDNF can elicit a regulatory role on APP processing enzymes remain elusive. Determining these pathways may allow for a more potent approach for future therapeutic treatments.

BDNF signaling is initiated upon its release from presynaptic dense core vesicles (Waterhouse & Xu, 2009), where it binds to its receptor, TrkB. This interaction between BDNF and TrkB results in receptor dimerization and kinase activation at the Tyr 512 site and Tyr 816 site (Kaplan & Miller, 2000; Patapoutian & Reichardt, 2001). Kinase autophosphorylation of TrkB results in the activation of phosphatidylinositol‐3‐kinase (PI3K)/Akt (Giese, 2013; Patapoutian & Reichardt, 2001; Vaynman et al., 2003, 2006). PI3K recruits Akt to the membrane, activating the protein at the Thr308 site or the Ser473 site. Upon activation, Akt phosphorylates glycogen synthase kinase (GSK)3β at the Ser9 site, which inhibits the protein (Cross et al., 1995). This inhibition of GSK3β is of particular interest as active GSK3β is thought to contribute to perturbed APP processing (Aplin et al., 1996; Rankin et al., 2007). Elevated GSK3β activity increases neuronal apoptosis and phosphorylates APP at the Thr 668 site, resulting in the precursor protein having a higher affinity for BACE1 cleavage (Aplin et al., 1996; Griffin et al., 2005; Hetman et al., 2000; Ly et al., 2013). Inhibition of GSK3β, via microdose lithium, results in reduced BACE1 expression and activity in the hippocampus of APP transgenic rats (Wilson et al., 2017). In our previous work, we demonstrated that BDNF treatments increased the phosphorylation status of Akt at the Ser473 site and resulted in higher GSK3β Ser9 phosphorylation in the hippocampus, while in the prefrontal cortex, BDNF treatment resulted in higher GSK3β Ser9 phosphorylation, indicating GSK3β inhibition. These findings are consistent with a previous study that showed that there is reduced GSK3β Ser9 phosphorylation in BDNF knockdown mice and with the administration of a TrkB antagonist (Gupta et al., 2014). Collectively, it is possible that the downstream effects of BDNF on BACE1 and ADAM10 may be regulated by changes in GSK3β signaling.

The purpose of this study was to determine whether GSK3β inhibition is a mechanism by which BDNF can elicit a regulatory role on BACE1 and ADAM10 activity. To this end, we used several models and targeted BDNF signaling with pharmacological antagonists to isolate pathways involved in the BDNF response. The data from this study provide critical information for future therapeutic strategies targeting the modulation of APP processing.

## 2 METHODS

### 2.1 Cell culture protocol

The neuronal SH‐SY5Y cell line, which has been approved by the Research Ethics Board at Brock University (No. 17‐397), was utilized to determine the mechanism/pathway through which BDNF modulates BACE1 activity. Undifferentiated SH‐SY5Y cells were cultured in Dulbecco's modified eagle medium (DMEM—cat. no. D6429—500 mL) containing 10% fetal bovine serum (FBS—cat. no. F1051—500 mL), 2% nonessential amino acids (NEAA‐cat. no. M7145—100 mL), and 1% penicillin/streptomycin (cat. no. P4333—100 mL) and left to proliferate until 80%–90% confluent. Cells were then seeded into 6‐well plates at a density of 100,000 cells/well. To initiate differentiation, cells were cultured with DMEM with 1% FBS, 2% NEAA and 1% penicillin/streptomycin, in conjunction with 10 μM of retinoic acid (Sigma Aldrich—cat. no. R2625). Differentiation took 7 days with the media and retinoic acid being changed every 2 days. Media was changed to fresh DMEM before the start of experiments. Following experiments, cells were lysed using 120 μL of NP40 cell lysis buffer (10 mL) supplemented with 34 μL phenylmethylsulfonyl fluoride and 50 μL protease inhibitor cocktail (Sigma; cat. no. 7626‐5G, cat. no. P274‐1BIL). Cells were scraped and collected in tubes, sonicated for two 20 s bouts on ice and immediately stored in a − 80°C freezer for future analysis. Each experiment was conducted with three replicates.

### 2.2 BDNF treatment dose–response in SH‐SY5Y cells

Cells were treated with 12.5, 25, 50, 75, or 100 ng/mL of recombinant mature BDNF (*n* = 4 in triplicate) for 1 h to determine the optimal dose. Recombinant mature BDNF (Millipore Sigma—cat. no. B3795) was reconstituted in dH_2_O and then diluted using DMEM with 1% FBS, 2% NEAA, and 1% penicillin/streptomycin. Collected cells were used for western blot analysis of markers of BDNF signaling.

### 2.3 BDNF treatment time‐course experiment in SH‐SY5Y cells

Cells were treated with the optimal dose of recombinant mature BDNF as established from the dose–response above (*n* = 4 in triplicate) for either 0, 30, 60, or 120 min to determine the time‐course of BDNF treatment and downstream signaling. Recombinant mature BDNF was reconstituted in dH_2_O and then diluted using DMEM with 1% FBS, 2% NEAA, and 1% penicillin/streptomycin. Collected cells were used for western blot and activity assay analysis.

### 2.4 BDNF treatment in SH‐SY5Y cells with wortmannin

To determine if BDNF‐induced regulation of BACE1 activity acts through the activation of Akt and subsequent GSK3β inhibition, cells were treated with 75 ng/mL of recombinant mature BDNF alongside the Akt‐specific inhibitor, wortmannin (1 μM—Selleck Chem—cat. no. S2758) for 1 h. Recombinant mature BDNF was reconstituted in dH_2_O, wortmannin was reconstituted in DMSO. Each solution was then diluted using DMEM with 1% FBS, 2% NEAA, and 1% penicillin/streptomycin prior to cell treatment. Collected cells were used for western blot and activity assay analysis.

### 2.5 Protein analysis

Protein concentration of the samples was determined using a bicinchoninic acid (BCA) assay (Sigma‐Aldrich—B9643, VWR—BDH9312). Samples were prepared to contain equal concentrations (1 mg/mL) of protein in 2 × Laemmli buffer and then placed in a dry bath at 100°C for 5 min and 10 μg of protein were loaded and separated on 10% SDS–PAGE gels for 90 min at 120 V. Proteins were then wet‐transferred onto nitrocellulose membrane at 100 V for 60 min. Membranes were blocked in Tris buffered saline/0.1% Tween 20 with 5% non‐fat powdered milk for 60 min at room temperature. The appropriate primary antibody was added and left to incubate on a shaker, at 4°C overnight. Antibodies against pro/mature BDNF (1:1000, ABCAM cat. no. 10819) CREB (1:1000, Cell Signaling cat. no. 9197), pCREB Ser133 (1:1000, Cell Signaling cat. no. 9196), TrkB (1:1000, Cell Signaling cat. no. 4603), pTrkB Tyr515 (1:1000, abcam cat. no. ab109684), APP (1:1000, Biolegend cat. no. SIG039152), pAPP Thr668 (1:1000, Cell Signaling cat. no. 6986S), BACE1 (1:500, Cell Signaling cat. no. 5606P), pBACE1 Ser498 (1:1000, Invitrogen cat. no. PA5‐118602), Akt (1:1000; Cell Signaling cat. no. 4685S), pAkt Ser473 (1:1000; Cell Signaling cat. no. 4058S), GSK3β (1:1000; Cell Signaling cat. no. 9315S), pGSK3β Ser9 (1:1000 Cell Signaling cat. no. 5558S), and ADAM10 (1:1000, ABCAM cat. no. 1997) were obtained and used during this study. Following primary incubation, the membrane was washed with Tris‐buffered saline/0.1% Tween 20 3 × 5 min and then incubated with the corresponding secondary antibody conjugated with horseradish peroxidase (Donkey anti‐rabbit IgG (H + L) 711‐035‐152, Goat anti‐mouse IgG (H + L) 115‐035‐003 Jackson Immunoresearch; West Grove, PA, 1:2000 ratio) for 60 min at room temperature. Signals were detected using enhanced chemiluminescence (ThermoFisher—cat. no. 50‐904‐9325**)** and then quantified by densitometry using a FluorChem HD imaging system (Alpha Innotech, Santa Clara, CA). A representative ponceau stain was measured and analyzed for each membrane to ensure equal loading (<10% variability across the membrane) (Sander et al., 2019).

## 3 ENZYMATIC ACTIVITY ASSAYS

### 3.1 BACE1 activity assay

BACE1 activity in the time course experiment was determined using a commercially available β‐secretase activity assay kit (Abcam—cat. no. Ab65357) as previously described (MacPherson et al., 2015; Yang et al., 2014). All samples were prepared at 0.50 μg/μL and 50 μL of sample was added to each well in duplicate, followed by 50 μL of 2 × reaction buffer and 2 μL of β‐secretase substrate. The plate was left to incubate in the dark at 37°C for 60 min and fluorescence was read using a spectrometer (SpectraMax M2; Molecular Devices) at excitation and emission wavelengths of 335 and 495 nm, respectively.

BACE1 activity in the in vitro wortmannin experiment (Figure 4) was determined in sample homogenates using a commercially available fluorigenic β‐secretase substrate (Sigma 565,758), active β‐secretase (Sigma S4195), and a β‐secretase inhibitor (Sigma S4562). Specifically, all samples were prepared at 1 μg/μL. 50 μL of sample was added to each well in duplicate, followed by 50 μL of 0.2 M sodium acetate assay buffer and 2 μL of β‐secretase substrate. The plate was left to incubate in the dark at 37°C for 60 min and fluorescence was read using a spectrometer (SpectraMax M2; Molecular Devices) at excitation and emission wavelengths of 350 and 490 nm, respectively.

### 3.2 ADAM10 activity assay

ADAM10 activity in SH‐SY5Y cells was determined using a commercially available ADAM10 activity assay kit (Anaspec; AS‐72226). Samples were homogenized and prepared as described in the BACE1 activity assay and 50 μL of sample was added to each well in duplicate, followed by 50 μL of the unique FRET (5‐FAM) ADAM10 substrate. The plate was left to incubate in the dark at 37°C for 30 min and fluorescence was read using a spectrometer (SpectraMax M2; Molecular Devices) at excitation and emission wavelengths of 490 and 520 nm, respectively.

### 3.3 In vitro supplement BACE1 and ADAM10 activity assay experiment

To examine the role of BDNF signaling through Akt on BACE1 activity an in vitro BDNF supplementation experiment was performed using a commercially available β‐secretase activity assay kit (Ab65357). Prefrontal cortex tissue homogenate was taken from male 19‐week‐old C57BL/6J mice, purchased from The Jackson Laboratory (Bar Harbor, Maine, USA), as previously described (Baranowski et al., 1985). Mice were anesthetized with a weight‐adjusted bolus intraperitoneal injection of sodium pentobarbital (5 mg/100 g body weight) and mice were euthanized through exsanguination (Baranowski et al., 1985). Prefontal cortex samples were homogenized (FastPrep®, MP Biomedicals, Santa Ana, CA) and extracted using 20 volumes of ice‐cold PBS. Samples were left to incubate on ice for 15 min and centrifuged at 10,000×*g* for 5 min at 4°C. The supernatant was collected, and a BCA assay was performed to determine protein concentration. Experimental protocols were approved by the Brock University Animal Care Committee (AUP: #20–07‐04) and are in compliance with the Canadian Council on Animal Care. All samples were prepared at 0.50 μg/μL and 50 μL of sample was added to each well in duplicate. Duplicate wells were then separated into four groups (*n* = 6 / group): (1) Control, (2) BDNF, (3) BDNF + ANA12 (TrkB antagonist), and (4) BDNF + wortmannin (Akt inhibitor). With exception to the control group, 5 μL of BDNF (75 ng/mL) was added to each sample, followed by either 5 μL of extraction buffer, 5 μL of ANA12 (5 μM), or 5 μL of wortmannin (1 μM). Once supplemented, 50 μL of 2 × reaction buffer and 2 μL of β‐secretase substrate was added to each well. The plate was left to incubate in the dark at 37°C for 30 min and fluorescence was read using a spectrometer (SpectraMax M2; Molecular Devices) at excitation and emission wavelengths of 335 and 495 nm, respectively.

### 3.4 Statistical analysis

Differences between total and phosphorylated protein content and enzyme activity were analyzed using a one‐way ANOVA. All data were tested for normal distribution via a Shapiro–Wilks test (*p* > 0.05). In cases where data were not normally distributed (Shapiro–Wilk test *p* < 0.05), data were logarithmically transformed. Data are expressed as means ± SEM with significance set at *p* < 0.05.

## 4 RESULTS

### 4.1 Dose–response and time course of BDNF treatment

**FIGURE 1:**
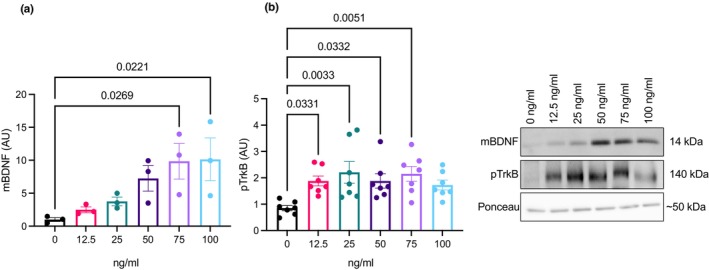
BDNF dose–response. (a) Changes in mBDNF (*n* = 3; technical triplicates). (b) Changes in TrkB phosphorylation (*n* = 7; technical triplicates). Data were analyzed using one‐way ANOVA and Tukey's post hoc test. Values are represented as mean ± SEM. Significance indicated by *p* < 0.05.

Prior to the time course treatment of BDNF, a dose–response experiment was performed to determine the optimal dose of BDNF that stayed within a normal physiological range. Our range was selected based on previous literature using BDNF in vitro (Xiong et al., 2013; Xu et al., 2019) and in vivo (Baranowski et al., 2021). A dose of 75 ng/mL of recombinant mature BDNF elicited a higher mBDNF content (*p* = 0.0269) as well as higher BDNF receptor phosphorylation (pTrkB Tyr 515; *p* = 0.01; Figure 1). Therefore, the remaining experiments were performed used the 75 ng/mL dose of recombinant mature BDNF.

**FIGURE 2:**
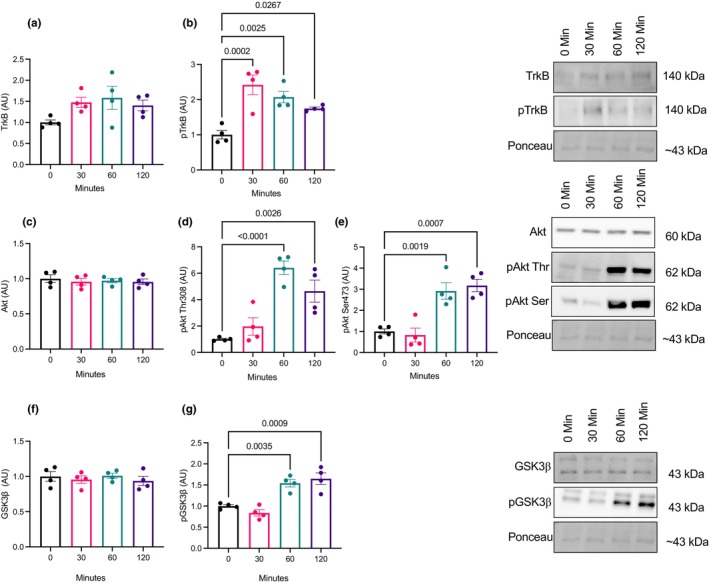
BDNF time course on BDNF signaling (*n* = 4; technical triplicates). (a, b) Total and phosphorylated TrkB protein content. (c–e) Total and phosphorylated Akt protein content at the Thr308 and Ser473 sites. (f, g) Total and phosphorylated GSK3β protein content. Data were analyzed using one‐way ANOVA and Tukey's post hoc test. Values are represented as mean ± SEM. Significance indicated by *p* < 0.05.

**FIGURE 3:**
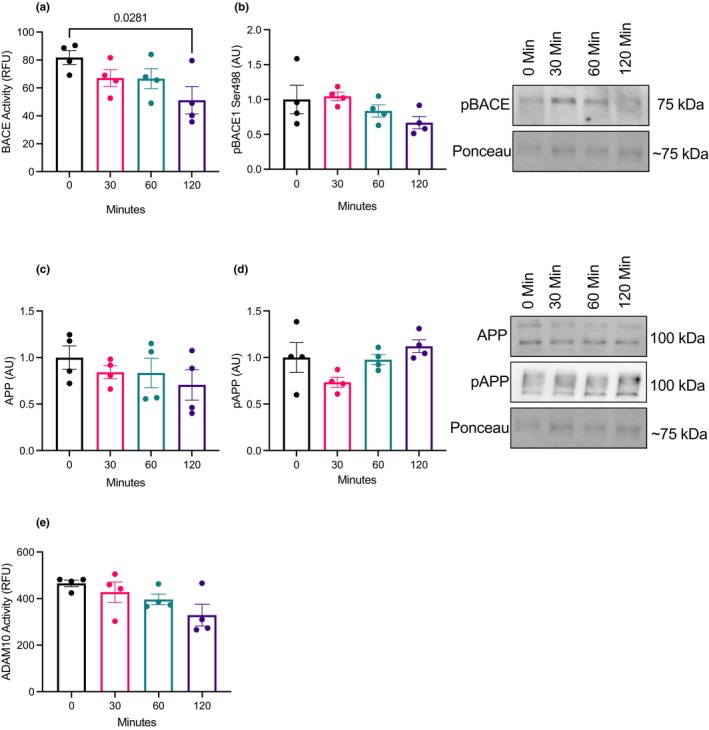
BDNF time course on APP processing (*n* = 4; technical triplicates). (a) BACE activity. (b) pBACE Ser498 protein content. (c, d) Total APP and pAPP protein content. (e) ADAM10 activity. Data were analyzed using one‐way ANOVA and Tukey's post hoc test. Values are represented as mean ± SEM. Significance indicated by *p* < 0.05.

A time course experiment was conducted to examine changes in BDNF signaling (TrkB, Akt, and GSK3β phosphorylation) as well as APP processing. There were no changes in total TrkB content (*p* = 0.29; Figure 2a), however, pTrkB Tyr 515 was elevated at 30, 60, and 120 min compared to the control (*p* = 0.0001; Figure 2b). There were no changes in total Akt content (*p* = 0.42; Figure 2c) however, pAkt Thr308 and Ser473 were elevated at 60 and 120 min (*p* = 0.0001; Figure 2d,e). Total GSK3β content did not change (*p* = 0.61; Figure 2f) however, pGSK3β Ser9 was elevated at 60 and 120 (*p* = 0.0001, Figure 2g). Total and phosphorylated APP content did not change (*p* = 0.37; Figure 3c,d). BACE1 activity was lower after 120 (*p* = 0.042; Figure 3a), while there were no changes in pBACE ser 498 site (*p* = 0.17; Figure 3b). There were no changes in ADAM10 activity with BDNF treatment (*p* = 0.13; Figure 3e).

### 4.2 In vitro BDNF treatment with wortmannin

**FIGURE 4:**
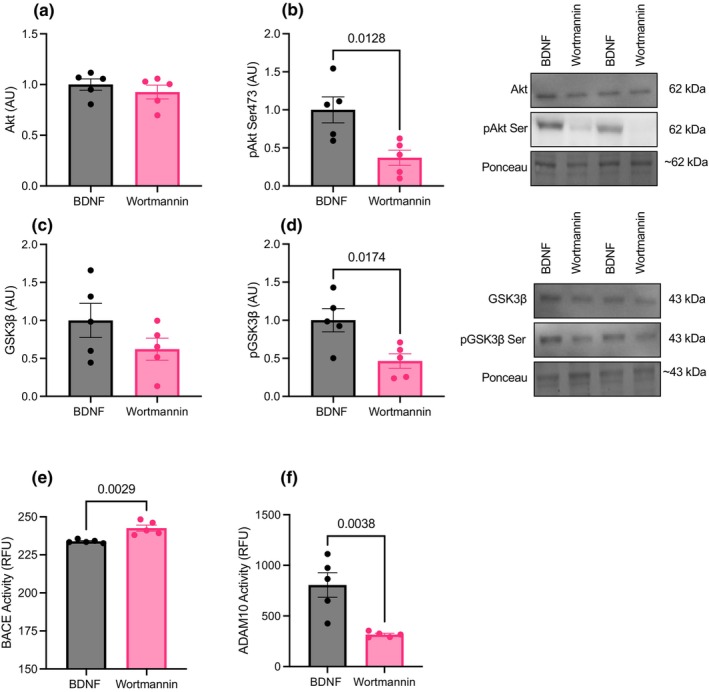
SH‐SY5Y cells treated with BDNF or BDNF+Wortmannin (*n* = 5; technical triplicates). (a, b) Total and phosphorylated Akt. (c, d) Total and phosphorylated GSK3β. (e) BACE activity. (f) ADAM10 activity. Data were analyzed using one‐way ANOVA and Tukey's post hoc test. Values are represented as mean ± SEM. Significance indicated by *p* < 0.05.

To further explore the potential role of GSK3β regulation on the BDNF‐induced modulation of APP processing, SH‐SY5Y cells were treated with either BDNF or BDNF + wortmannin, a selective Akt inhibitor. Upon activation, Akt phosphorylates GSK3β at the Ser9 site, which inhibits the protein (Cross et al., 1995); therefore, inhibiting Akt effectively prevents GSK3β inhibition. Cells treated with BDNF + wortmannin had no differences in total Akt (*p* = 0.42; Figure 4a) or total GSK3β (*p* = 0.19; Figure 4c) however, pAkt Ser473 (*p* = 0.01; Figure 4b) and pGSK3β Ser9 (*p* = 0.01; Figure 4d) was lower compared to cells treated with BDNF. In the same model, SH‐SY5Y cells treated with BDNF + wortmannin had higher BACE1 activity (*p* = 0.002; Figure 4e) and lower ADAM10 activity (*p* = 0.003; Figure 4f) compared to cells treated with BDNF alone.

### 4.3 In vitro supplemented BACE1 and ADAM10 activity assay

**FIGURE 5:**
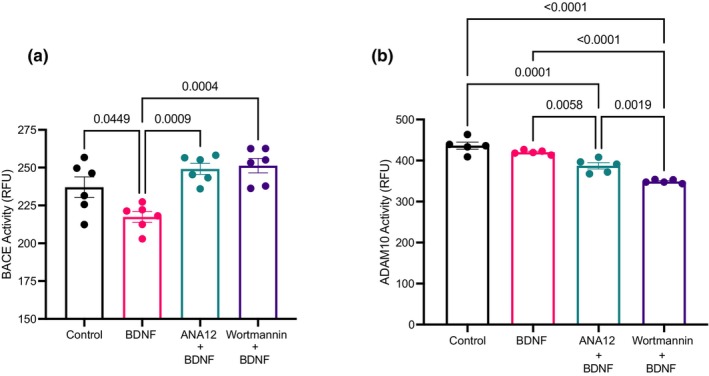
In vitro assay (*n* = 5; technical triplicates). (a) BACE1 activity. (b) ADAM10 activity. Data were analyzed using one‐way ANOVA and Tukey's post hoc test. Values are represented as mean ± SEM. Significance indicated by *p* < 0.05.

Tissue homogenate samples from the prefrontal cortex of male C57BL/6J mice were treated with BDNF and then supplemented with either the TrkB antagonist, ANA‐12 or with wortmannin immediately before a BACE1 activity assay to determine whether these pathways are necessary for the BDNF‐mediated alterations in BACE1 activity. This type of in vitro experiment has been established to examine BACE1 activity (Devi & Ohno, 2012) and allows for examination of enzyme activity in the presence of multiple cell types (i.e., neurons, astrocytes, glial cells, etc.) providing a slightly more physiological model than neuronal cell culture. BDNF supplementation in prefrontal cortex homogenate lowered BACE1 activity compared to all groups (*p* = 0.003; Figure 5a). This reduction in BACE1 activity was attenuated when the homogenate was treated with both BDNF and ANA‐12 (*p* = 0.0009) and when treated with BDNF and wortmannin (*p* = 0.0004). BDNF supplementation did not change ADAM10 activity in prefrontal cortex homogenate (*p* = 0.25; Figures 4b and 5); however, there was lower ADAM10 activity when the homogenate was treated with both BDNF and ANA‐12 (*p* = 0.0001) and with BDNF and wortmannin (*p* = 0.0001) compared to control and BDNF groups.

## 5 DISCUSSION

This study provides a novel examination of a potential mechanism by which BDNF regulates APP processing. The results demonstrate a dose and time course response to BDNF treatment in SH‐SY5Y cells. Specifically, we show that BDNF treatment reduces BACE1 activity and that this likely occurs through the activation of Akt and subsequent inhibition of GSK3β. This is the first study to demonstrate a regulatory role of BDNF on BACE1 activity in a neuronal cell line. Using pharmacological antagonists, we narrow in on Akt activation and potentially GSK3β inhibition being critical components of BDNF‐induced modulation of BACE1 and ADAM10 activity. Specifically, we show with in vitro models, samples treated with BDNF and wortmannin, an Akt inhibitor, no longer reduce BACE1 activity. Further, treatment with ANA‐12 and wortmannin in an in vitro model demonstrated lower ADAM10 activity, highlighting BDNF signaling and Akt activation as critical components in ADAM10 regulation. The results from this study have clinical implications with respect to the development of novel therapeutic strategies that target APP modulation.

Here, we demonstrate that BDNF treatment of SH‐SY5Y neuronal cells results in lower BACE1 activity. This adds to our previous work where BDNF treatment of brain explant tissue as well as peripheral injections of BDNF resulted in lower BACE1 activity (Baranowski et al., 1985, 2021). However, BDNF treatment in SH‐SY5Y cells did not alter ADAM10 activity, a result contrary to previous in vivo findings from our lab (Baranowski et al., 1985) and in vitro findings from others (Nigam et al., 2017). Previous work from our lab demonstrated that C57BL/6J mice treated daily with BDNF elevated ADAM10 activity in the prefrontal cortex and hippocampus (Baranowski et al., 1985), while Nigam and colleagues treated SH‐SY5Y cells with BDNF for 3 days and started before the cells were fully differentiated (Nigam et al., 2017). The difference in protocol and treatment duration may explain the discrepancy with the acutely treated cells in this study. Despite our evidence that BDNF can modulate BACE1 activity, the exact mechanism(s) behind this effect remain elusive. To interrogate this mechanism, we examined downstream markers of BDNF signaling. As anticipated, phosphorylation of BDNF's receptor, TrkB, was elevated after 30 min of BDNF treatment and remained elevated up to 120 min indicating that the BDNF signaling cascade was activated. We also demonstrate that the downstream target, Akt, had elevated phosphorylation at the Thr308 site and Ser473 site after 60 min, which is in line with our pTrkB Tyr515 data. Following 60 min of treatment, BDNF also resulted in the elevation of pGSK3β Ser9, indicating inhibition of GSK3β. Active GSK3β phosphorylates APP at the Thr 668 site, resulting in the precursor protein having a higher affinity to BACE1 cleavage (Aplin et al., 1996; Griffin et al., 2005; Hetman et al., 2000; Ly et al., 2013). Given the connection between GSK3β and APP processing, we suspected that BDNF signaling is modulating BACE1 activity through GSK3β inhibition.

To test this hypothesis, we treated SH‐SY5Y cells with BDNF or BDNF and wortmannin, a selective PI3K/Akt inhibitor. Treating SH‐SY5Y cells with wortmannin lowered Akt Ser 473 phosphorylation and subsequently lowered GSK3β Ser 9 phosphorylation, preventing GSK3β inhibition. In our previous experiment, we demonstrate that BDNF treatment lowered BACE1 activity (Figure 3a) and did not impact ADAM10 activity (Figure 3e), however, when cells were treated with BDNF and wortmannin, BACE1 activity was elevated and ADAM10 activity was lowered compared to cells that were treated with BDNF alone. Nigam and colleagues demonstrated that chronic BDNF treatment reduced ADAM10 activity in SH‐SY5Y cells and therefore speculated that BDNF‐induced regulation of APP processing was driven through elevated ADAM10 regulation (Nigam et al., 2017). However, the present study provides a line of evidence to suggest that acute effects of BDNF treatment on APP processing is predominantly through the regulation of BACE1 activity. Specifically, we are showing that BDNF treatment blunts BACE1 activity, whereas ADAM10 activity is only affected in the presence of inhibited BDNF signaling. This indicates that BDNF signaling plays a role in maintaining ADAM10 activity rather than elevating it. With these findings, we speculate that by inhibiting Akt phosphorylation via wortmannin, BDNF was no longer able inhibit GSK3β and subsequently prevented a regulatory role on APP processing.

Although in vitro experiments are ideal for elucidating cellular mechanisms, a limitation of using the SH‐SY5Y neuronal cell line is a lack of translatability as it only represents a single cell type in isolation. As the brain is composed of a multitude of cell types, such as glial, microglial, and astrocytes (Zeisel et al., 2015) examining the effects of BDNF on BACE1 regulation through GSK3β signaling needs to be performed in a model that accurately represents the distribution of cell types found in the brain. To account for this distribution of cell types and to determine if the activation of TrkB and the downstream Akt pathway is required for BDNF to reduce BACE1 activity, mouse prefrontal cortex tissue homogenate was treated with BDNF, a TrkB antagonist (5 μM ANA‐12), and wortmannin (1 μM). These results demonstrated that BDNF treatment reduced BACE1 activity compared to control, which is in line with our previous cell culture results. With the administration of BDNF + ANA‐12, the reduction in BACE1 activity is ablated, which indicates that BDNF binding to its receptor, specifically TrkB, is required for the reductions in BACE1 activity. Mouse prefrontal cortex homogenate treated with BDNF and wortmannin had higher BACE1 activity compared to BDNF treatment alone (Figure 5). Wortmannin is a specific PI3K inhibitor which effectively prevents the activation of Akt, an action necessary for the inhibition of GSK3β. These results suggest that Akt activation is necessary for BDNF‐induced reductions in BACE1 activity. Collectively, these experiments narrow in on the mechanism by which BDNF can modulate BACE1, specifically, BDNF must bind to its receptor TrkB to activate the PI3K/Akt pathway and is potentially mediated through GSK3β signaling. Corroborating the time‐course experiment, acute BDNF treatment did not alter ADAM10 activity. However, when TrkB and Akt were inhibited, there was a reduction in ADAM10 activity, highlighting the necessity of BDNF signaling to maintain ADAM10. This study confirms a link between BDNF and ADAM10 activity but the mechanism connecting them has yet to be elucidated.

A limitation of this study is the indirect method of preventing GSK3β phosphorylation at the Ser 9 site. Wortmannin is a widely used PI3K inhibitor, which has been shown to reduce Akt activation, indirectly preventing GSK3β Ser9 phosphorylation (Powis et al., 1994). However, there are other signaling pathways downstream of Akt activation and therefore it can only be speculated that GSK3β is a regulator of BACE1 activity. Currently, there are no known pharmacological GSK3β activators, an intervention necessary to link BDNF‐induced regulation of BACE1 with GSK3β regulation. As an alternative, future research should examine the effects of BDNF treatment in cells transfected with the GSK3β S9A mutation. This mutation would prevent GSK3β inhibitory phosphorylation from occurring, allowing the determination if BDNF‐induced GSK3β inhibition is necessary for BACE1 regulation.

## 6 CONCLUSIONS

This study provides novel findings regarding the regulation of BDNF on APP processing. Specifically, we demonstrate that BDNF treatment reduced BACE1 activity and elevated GSK3β inhibition in an SH‐SY5Y cell line. Our loss‐of‐function experiments in murine prefrontal cortex homogenate and SH‐SY5Y cells narrow in on the PI3K/Akt pathway being critical in BDNF‐induced reductions in BACE1 activity and maintaining ADAM10 activity. These results further support the hypothesis that BDNF is altering BACE1 activity via GSK3β inhibition. Although preliminary, these finding have clinical implications in designing future therapeutic strategies for regulating BACE1 and ADAM10 activity. Elucidating GSK3β activity as a mediator of BDNF‐induced BACE1 modulation allows for a more targeted approach with therapeutic interventions. For example, these findings can drive new research to explore the use of GSK3β inhibitors to target BACE1 activity in conjunction with BDNF or BDNF mimetics.

## AUTHOR CONTRIBUTIONS

The authors confirm contribution to the paper as follows: study conception and design: R.E.K.M., B.J.B., P.J.L., A.M., V.A.F.; data collection: B.J.B., A.M., R.M.; analysis and interpretation of results: B.J.B., R.M.; draft manuscript preparation: B.J.B., R.M. All authors reviewed the results and approved the final version of the manuscript.

## FUNDING INFORMATION

This work was supported by the Natural Sciences and Engineering Research Council of Canada Discovery Grant (Grant no. RGPIN‐2017‐03904) and the Alzheimer's Society of Brant, Haldimand Norfolk, Hamilton Halton and to REK MacPherson. BJ Baranowski was supported by NSERC CGSD. A. Mohammad was supported by NSERC USRA.

## CONFLICT OF INTEREST STATEMENT

The authors declare that the research was conducted in the absence of any commercial or financial relationships that could be construed as a potential conflict of interest.
